# The rice *ALS3* encoding a novel pentatricopeptide repeat protein is required for chloroplast development and seedling growth

**DOI:** 10.1186/s12284-015-0050-9

**Published:** 2015-04-09

**Authors:** Dongzhi Lin, Xiaodi Gong, Quan Jiang, Kailun Zheng, Hua Zhou, Jianlong Xu, Sheng Teng, Yanjun Dong

**Affiliations:** Development Center of Plant Germplasm Resources, College of Life and Environment Sciences, Shanghai Normal University, Shanghai, 200234 China; Present address: Institute of Genetics and Developmental Biology Chinese Academy of Sciences, No.1 West Beichen Road, Chaoyang District, Beijing, 100101 China; Present address: Agricultural Faculty, Hokkaido University, Sappro, 060-0817 Japan; Institute of Crop Science, National Key Facility for Crop Gene Resources and Genetic Improvement, Chinese Academy of Agricultural Sciences, Beijing, 10081 China; Institute of Plant Physiology and Ecology, Shanghai Institute for Biological Sciences, Chinese Academy of Sciences, Shanghai, 200032 China

**Keywords:** Albino, Chloroplast development, Lethality, Rice, Pentatricopeptide repeat (PPR) proteins

## Abstract

**Background:**

Pentatricopeptide repeat (PPR) proteins play essential roles in modulating the expression of organelle genes and have expanded greatly in higher plants. However, molecular mechanisms of most rice PPR genes remain unclear.

**Results:**

In this study, a new rice PPR mutant, *asl3* (*albinoseedlinglethality3*) exhibits an albino lethal phenotype at the seedling stage. This albino phenotype was associated with altered photosynthetic-pigment and chloroplast development. Map-based cloning showed that *ASL3* encodes a novel rice PPR protein with 10 tandem PPR motifs, which localizes to the chloroplast. *ASL3* showed tissue-specific expression, as it was highly expressed in the chlorenchyma, but expressed at much lower levels in roots and panicles. RNAi of *ASL3* confirmed that *ASL3* plays an essential role in the early development and chloroplast development in rice. Moreover, expression analysis revealed that the *asl3* mutation severely affected the transcriptional levels of important genes associated with plastid translation machinery and photosynthesis, which may impair photosynthesis and finally led to the seedling death in *asl3* mutant. These results evidenced the important role of *ASL3* in the early development of rice, especially chloroplast development.

**Conclusions:**

The ASL3 gene encoded a novel chloroplast-targeted PPR protein with 10 tandem PPR motifs in rice. Disruption of the ASL3 would lead to a defective chloroplast and seedling lethality, and affected expression levels of genes associated with chloroplast development and photosynthesis at early leaf stage of rice.

**Electronic supplementary material:**

The online version of this article (doi:10.1186/s12284-015-0050-9) contains supplementary material, which is available to authorized users.

## Background

The pentatricopeptide repeat (PPR) family was first recognized from the *Arabidopsis* thaliana genome sequence (Small and Peeters 2000). The PPR proteins are characterized by a degenerate motif of 35 amino acids that can be repeated up to 30 times within a single protein. They are predicted to comprise an array of α helices (Small and Peeters 2000), placing them in the ‘a-solenoid’ superfamily that includes tetratricopeptide repeat (TPR) proteins, ankyrin repeat proteins, HEAT domain proteins and Puf domain RNA-binding proteins. The PPR proteins can be separated into two major subfamilies based on the nature of their PPR motifs and into several smaller subclasses based on their C-terminal domain structure (Lurin et al. 2004; O’Toole et al. 2008). Additionally, the genomes of the parasitic protozoan *Trypanosoma brucei*, yeast, drosophila, and human are predicted to contain only 28, 5, 2, and 6 PPR genes, respectively (Lurin et al. 2004; O’Toole et al. 2008; Asano et al. 2013). However, the PPR family has expanded greatly in higher plants, with 466 members in *Arabidopsis* and 477 members in rice, suggesting that PPR protein genes diversified during the evolution of the land plants (Lurin et al. 2004; Schmitz-Linneweber and Small 2008).

To date, all confirmed physiological roles of PPR proteins are within mitochondria or chloroplasts (Schmitz-Linneweber and Small 2008). Most PPR proteins act as sequence-specific RNA binding factors that are involved in the post-transcriptional regulation of organelle gene expression (Delannoy et al. 2007). In chloroplasts, some PPR proteins have been found to participate in RNA splicing (Schmitz-Linneweber et al. 2006; de Longevialle et al. 2007; Ichinose et al. 2012), RNA processing (Meierhoff et al. 2003; Hattori et al. 2007), RNA editing (Kotera et al. 2005; Okuda et al. 2007; Chateigner-Boutin et al. 2008; Cai et al. 2009; Yu et al. 2009; Zhou et al. 2009; Tseng et al. 2010; Sosso et al. 2012), translation (Williams and Barkan, 2003; Tavares-Carreón et al. 2008), and RNA stability (Yamazaki et al. 2004; Pfalz et al. 2009). Despite the few PPR proteins of which molecular functions have been characterized in detail, a lot of work still to be done is to identify the functions of the other PPR proteins in plant development, especially in rice.

Functional studies of rice PPR proteins remain very sparse and a mutation in a PPR gene usually has a strong phenotypic effect. *OsPPR1*, including 11 PPR motifs, is the first report on the rice PPR protein required for the chloroplast biogenesis (Gothandam et al. 2005). Antisense transgenic strategy was used to suppress the expression of *OsPPR1* and the resulted transgenic rice showed the typical phenotypes of chlorophyll-deficient mutants, albinism and lethality. Another rice PPR protein, YSA, with 16 PPR motifs, is required for chloroplast development in early seedling leaves, and disruption of its function causes a seedling stage-specific albino phenotype (Su et al. 2012). *OsV4* encodes a PPR protein targeted to the chloroplast, which is essential for chloroplast development during the early leaf stage under cold stress (Gong et al. 2014). The *osv4* mutant exhibits albino phenotype at a restrictive temperature (20°C) before the 4-leaf stage and gradually turned green as the leaf number rose, but it is always green at 32°C.

Here, we isolated a new rice albino seedling lethal mutant, *asl3*, which develops albino leaves before the 3-leaf stage, thereafter died. Map-based cloning and further analysis revealed that *ASL3* encodes a novel PPR protein containing 10 tandem PPR motifs, whose biological action is required for early chloroplast development and photosynthesis in rice.

## Results

### Characterization of the *asl3* mutant

**Figure 1:**
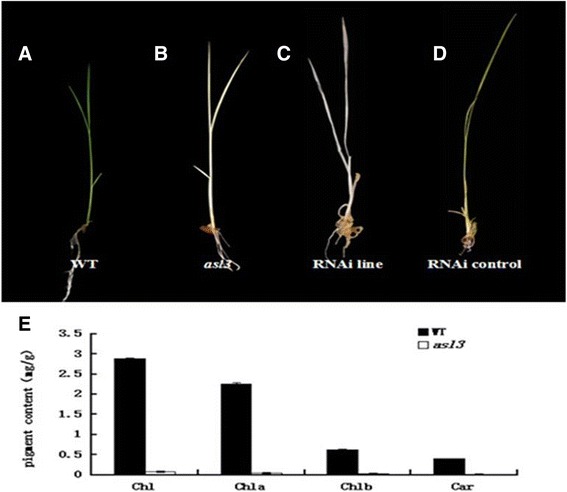
**Characterization of the** ***asl3*** **mutants at 3-leaf stage: (A) WT plants (Jiahua 1) (B)** ***asl3*** **mutant plants; (C) RNAi transgenic line transformed with pTCK303-dsRNAiASL3; (D) RNAi control; (E) The pigment contents in leaves at 3-leaf stage in** ***asl3*** **mutants are much lower than that in WT plant.** Chlorophyll a (Chla), chlorophyll b (Chlb), total chlorophyll (Chl) and carotenoid (Car).

The *asl3* mutant was a lethal mutant isolated from a ^60^Co-irradiated population of japonica variety Jiahua1 (WT). All leaves of *asl3* seedlings exhibited an albino phenotype at the seedling stage (Figure 1A,B), and the seedlings did not survive past the 4-leaf stage because of no photosynthesis to provide nutrition. In addition, the accumulation of chlorophyll (Chl) a, b and carotenoid (Car) were negligible in the *asl3* seedlings (Figure 1E), which was consistent with the albino phenotype.

**Figure 2:**
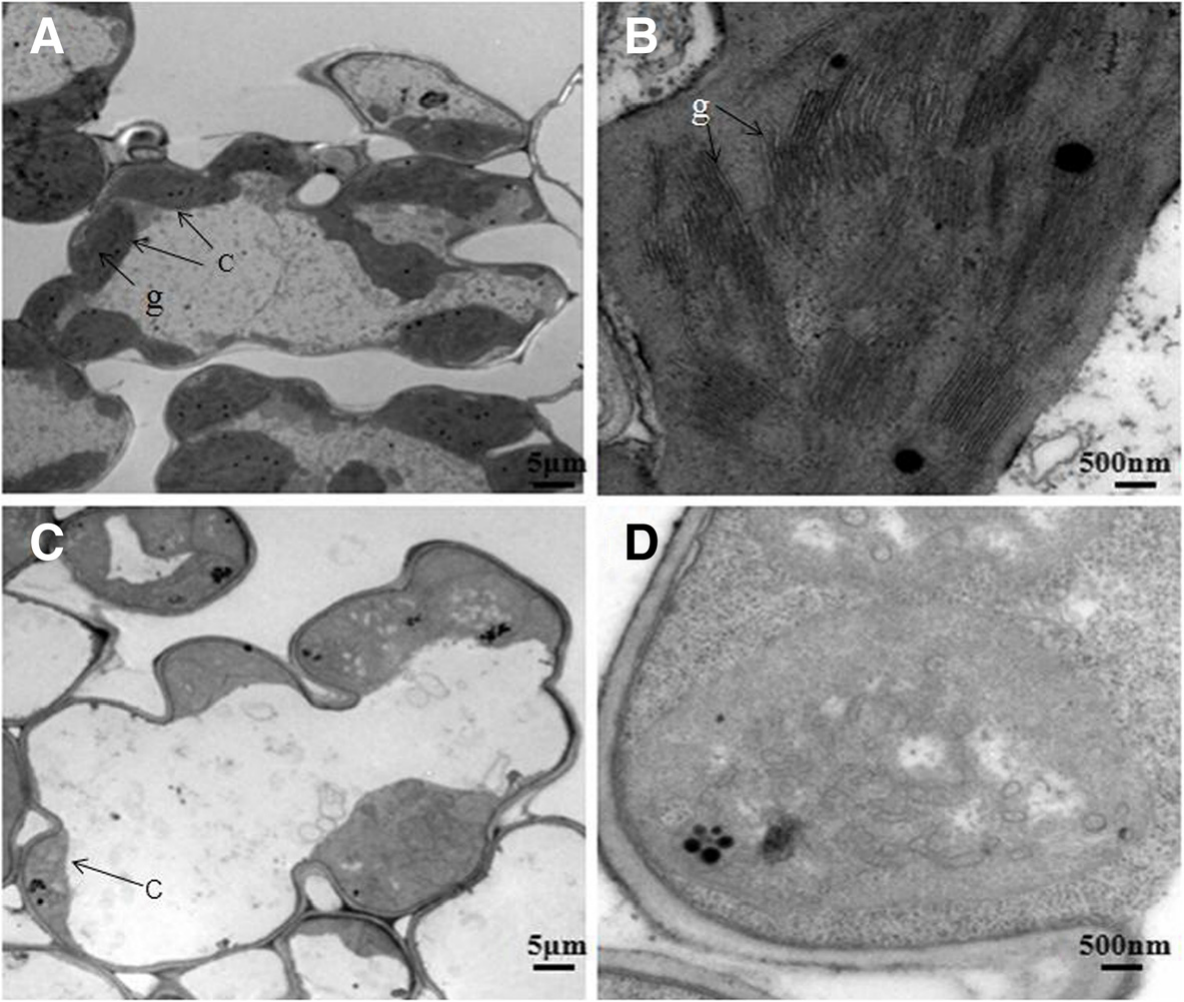
**Transmission electron microscopy of chloroplasts in expanded third leaves: (A) The cell of wild type; (B) An intact chloroplast in the wild type cell; (C) The cell of an** ***asl3*** **mutant; (D) An abnormal chloroplast in the** ***asl3*** **mutant cell.** c, chloroplast; g, grana stack.

To investigate chloroplast development in *asl3* mutant, the ultrastructure of chloroplasts at 3-leaf stages were examined by transmission electron microscopy (TEM). As expected, the unabridged chloroplast was found in all WT plants and the grana stacks were dense and well structured (Figure 2A,B), whereas chloroplast did not display the usual architecture and had no observable grana lamella stacks in *asl3* mutant (Figure 2C,D). These observations indicate that the *asl3* mutation results in abnormal development of the chloroplasts.

### Map-based cloning of the *ASL3* gene

To elucidate the molecular mechanism responsible for the phenotype of *asl3* mutant, map-based cloning was performed to identify the *ASL3* locus. Due to no seeds could be obtainable in homozygous mutants because of the seedling-lethality, the crosses of the heterozygous *ASL3/asl3* plants with *indica* cultivar Pei’ai64S were conducted to generate a segregation population for gene mapping. The F_1_ plants (*ASL3/ASL*3: *ASL3/asl3* = 1:1) from the crosses were all normal green; however, segregation occurred in the F_2_ plants selfed from the heterozygous F_1_ plants (*ASL1/asl1*) in the proportion of 3:1 (green: albino = 313:98; χ2 = 0.21; *P* > 0.05), indicating that this mutation in *asl3* plants is a single recessive locus.

**Figure 3:**
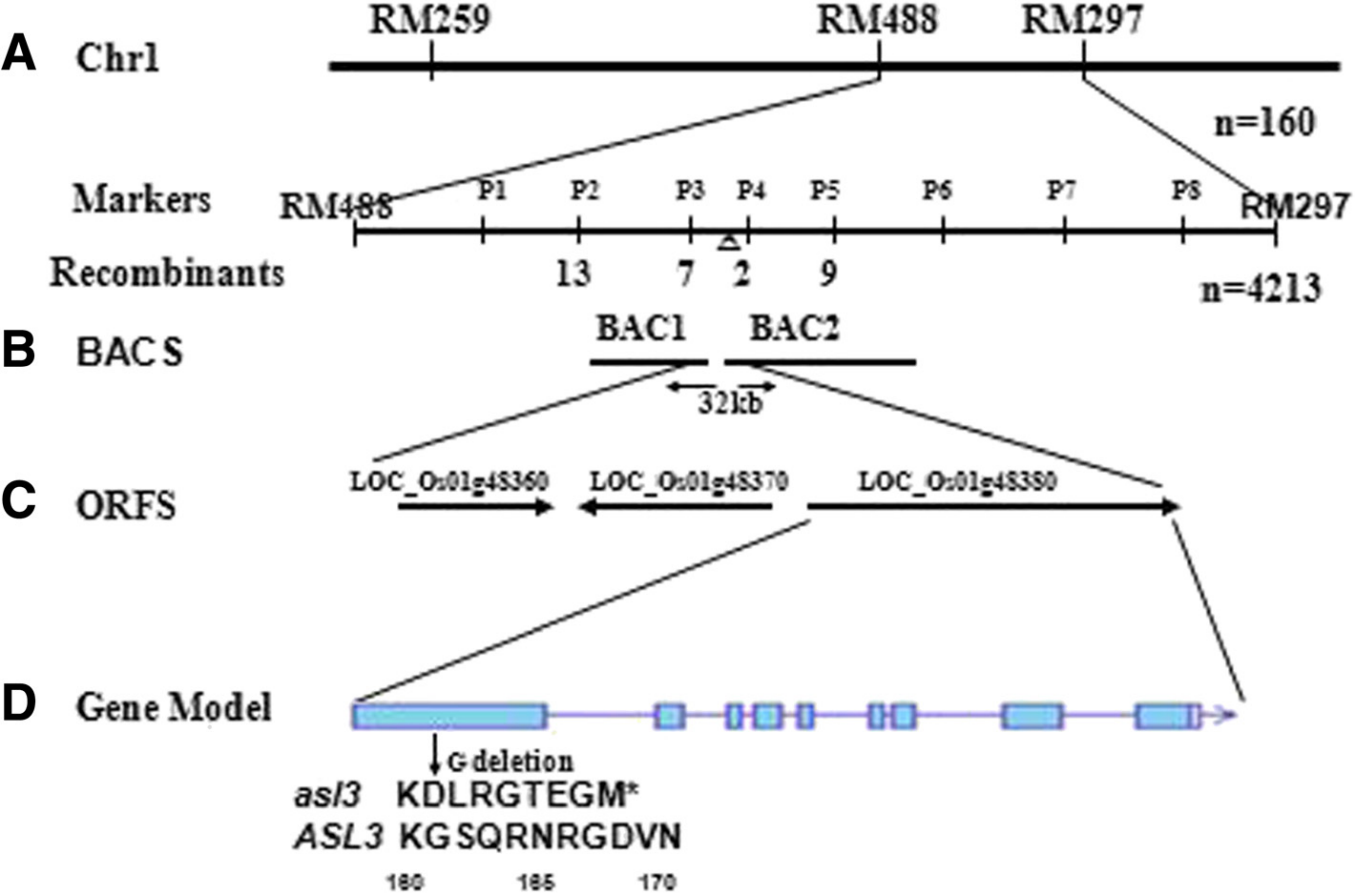
**Map-Based cloning of** ***ASL3*** **: (A) The** ***ASL3*** **locus was initially mapped to a region between markers RM488 and RM297 on the long arm of rice chromosome 1 (Chr.1) with 160 recessive individuals; (B) Fine mapping of** ***ASL3*** **between BAC1 (AP008207.2) and BAC2 (AP006867) within a 32-kb region by the markers P3 and P4 using 4,213 mutant individuals; (C) Diagram of the predicted ORFs and the mutation site; (D) Gene model of** ***ASL3*** , a 1-bp deletion (G*) in ***LOC_Os01g48380*** **results in a premature stop codon.**

The *ASL3* locus was initially mapped to the long arm of chromosome 1(Chr1) between the molecular markers RM488 and RM297 by analyzing 160 mutant individuals (Figure 3A). Then a larger F_2_ population with 4213 mutant individuals was used for fine mapping. Eight InDel markers (P1 → P8) were developed between RM488 and RM297. The *ASL3* locus was further narrowed down to a 32-kb region between P3 and P4 (Figure 3B), which included 3 putative open reading frames (ORFs) (http://rice.plantbiology.msu.edu) (Figure 3C). All putative ORFs were sequenced and a 1-bp deletion (G*) was found in *LOC_Os01g48380*, causing a premature stop codon (Figure 3D).

### Knockdown of *ASL3* displays the lethal phenotypes

**Figure 4:**
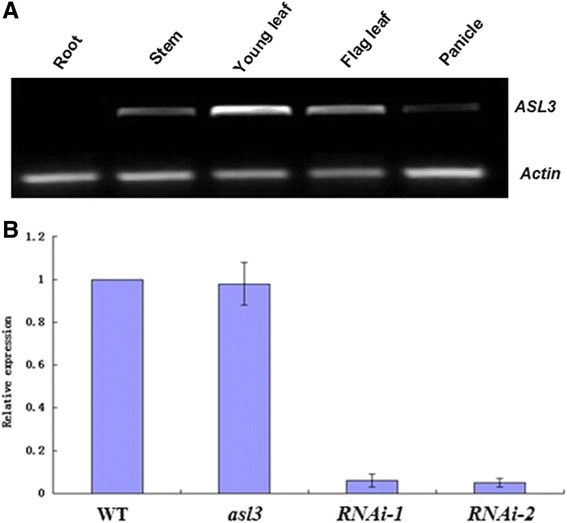
**Expression analysis of** ***ASL3:*** **(A) RT–PCR analysis of** ***ASL3*** **in root, young stem, young leaf, flag leaf and panicle of WT** ***.*** Rice *Actin* gene was used as a control; **(B)** Transcript levels of *ASL3* in top leaves sampled from WT, *asl3* mutant, RNAi lines at 3-leaf-stage.

To understand whether the function-loss of *ASL3* is responsible for the lethal phenotype in mutant, RNA interference (RNAi) technology was used to suppress *ASL3* expression in WT plants. A gene-specific fragment of *ASL3* was cloned into an RNAi vector and transgenic plants were generated via *Agrobacterium*–mediated transformation. Resultantly, fifty-one RNAi lines showed the same albino phenotypes as in the *asl3* mutant (Figure 1C). Further, two RNAi transgenic plants with albino phenotypes were selected for measurement of *ASL3* transcript. The *ASL3* transcripts of RNAi lines were significantly lower than that of the WT plants (Figure 4B). These results confirmed that RNAi of *ASL3* could mimic the phenotypes of the *asl3* mutant.

### Characterization of the predicted ASL3

Sequence analysis of the genomic DNA and cDNA revealed that the *ASL3* gene is comprised of 9 exons and 8 introns and encoded a polypeptide of 994 amino acids with a calculated molecular mass of 113.05 kD. The functional domain analysis using TPRpred (Karpenahalli et al. 2007) reveals that ASL3 is a PPR protein containing 10 PPR motifs. Although bioinformatics (http://rice.plantbiology.msu.edu) shows that there is another transcript, we haven’t detected it by RT-PCR method using specific primers (data not shown).

**Figure 5:**
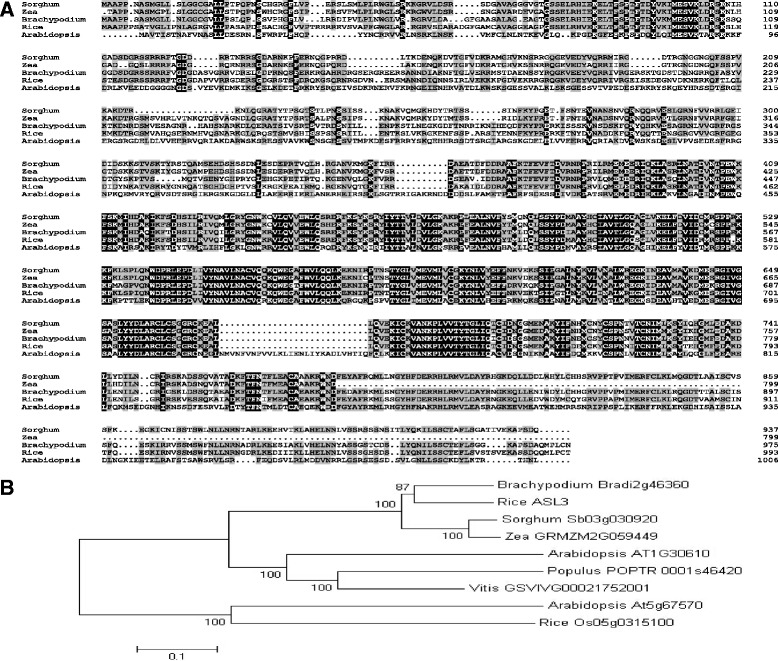
**Phylogenic analysis of ASL3 Protein: (A) Amino acid sequence alignment of its homologs** ***Arabidopsis thaliana*** **,** ***Brachypodium distachyon*** **,** ***Sorghum bicolor*** **and** ***Zea mays*** **.** Amino acids fully or semi-conserved are shaded black and gray, respectively; **(B)** Homologous proteins similar to ASL3 were used to obtain a phylogenetic tree with the program Mega5.1, which was bootstrapped over 1,000 cycles. Significance values above a 50% cutoff threshold are indicated near the relative branches.

Orthologs of ASL3 from *Arabidopsis thaliana*, *Brachypodium distachyon*, *Sorghum bicolor* and *Zea mays* were found in the NCBI database. ASL3 has 42–74% amino acid sequence identity to the four characterized orthologs. Among these, ASL3 exhibits maximum sequence similarity with protein in *Brachypodium distachyon*, with 74% amino acid identity and it shared 42% peptide identity with protein from *Arabidopsis* (Figure 5A). These data indicated that the ASL3 protein is highly conserved in higher plants. Eight related proteins were used to investigate the relationship between ASL3 homologs in evolutionary history. As shown in Figure 5B, they could be divided into two groups: (1) the orthologs proteins from both monocots and dicots are divided clearly into two subgroups; (2) another two paralogous proteins from rice and *Arabidopsis* forms another group.

### Subcellular localization of ASL3

**Figure 6:**
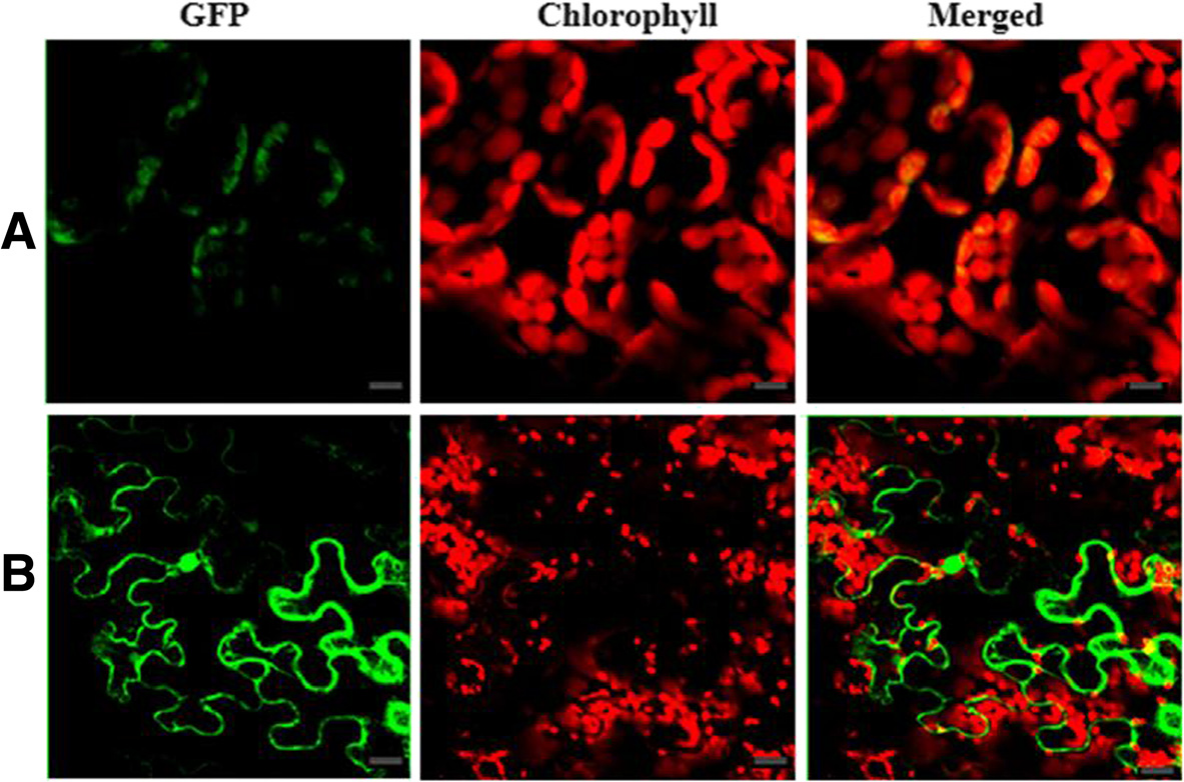
**Subcellular localization of the ASL3’ protein: (A) A tobacco mesophyll cell expressing ASL3–GFP; (B) A tobacco epidermal cell expressing GFP alone The scale bar represents 20 μm.**

The ASL3 protein was predicted to localize to chloroplasts according to ChloroP (http://www.cbs.dtu.dk/services/ChloroP/) and TargetP (http://www.cbs.dtu.dk/services/TargetP/). To examine the actual subcellular localization of ASL3, the cDNA fragment encoding the N-terminal region (amino acids 1–249) of the ASL3 was amplified from WT plants and introduced into the N-terminal of the GFP gene in the expression vector pMON530-GFP. The pMON530:CaMV35S:ASL3-GFP plasmid was introduced into tobacco cells using Agrobacterium-mediated infection method. Meanwhile, empty GFP vector was used as a control. As a result, the green fluorescent signals of ASL3-GFP fusion protein perfectly overlapped with chloroplast autofluorescence in transformed tobacco mesophyll cells (Figure 6A). By contrast, the epidermis cells transformed with the empty GFP vector without a specific targeting sequence had green fluorescent signals in both plasma membrane, cytoplasm and the nucleus. Thus, these findings suggest that ASL3 is localized to the chloroplast (Figure 6B).

### Expression pattern of *ASL3 gene*

Reverse transcription PCR (RT-PCR) was performed to examine the expression pattern of *ASL3*. Resultantly, a significantly high level of expression was detected in stems, young leaves and flag-leaves, but a very limited amount of the transcript was detected in roots and panicles (Figure 4A), suggesting that the ASL3 mainly functions in the chlorenchyma. Interestingly, the transcripts of *ASL3* had no obvious change in *asl3* plants (Figure 4B), showing that the 1-bp deletion could not affect its transcriptional expression in *asl3* plants.

### The transcript expressions of related genes in the *asl3* mutant

**Figure 7:**
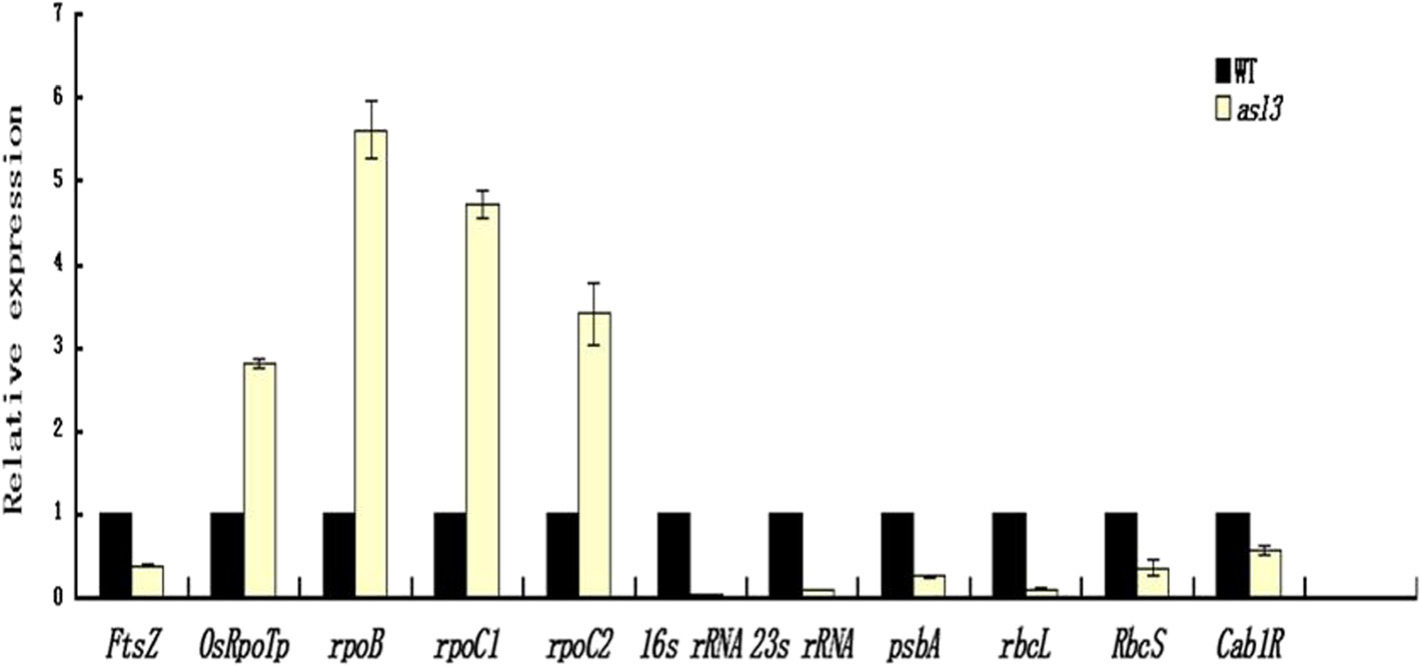
**Expression analysis of genes associated with chlorophyll biosynthesis, photosynthesis, or chloroplast development by real-time PCR.** The relative expression level of each gene was normalized using *Actin* as an internal control. The expression level of each gene at the three-leaf stage in Jiahua1 was set as 1.0 and other samples were calculated accordingly. Error bars (SDs) are based on three independent experiments.

To assess the possibility that the impaired chloroplasts in *asl3* mutant may be reflected at the level of related gene expression, we examined the transcription levels of genes associated with photosynthesis and chloroplast development both in the *asl3* mutant and WT plant by qPCR analysis. The photosynthesis-associated transcripts of plastid genes, *psbA* (encoding a reaction center polypeptides) and *rbcL* (encoding the large subunit of Rubisco), the nuclear genes *RbcS* (encoding the small subunit of Rubisco, Kyozuka et al. 1993) and *Cab1R* (encoding the light harvesting Chla/b-binding protein of PSII), were significantly suppressed in the *asl3* mutant, which may impair photosynthesis ability and finally led to the seedling lethality in mutant (Figure 7).

As for chloroplast-development associated transcripts, the levels of PEP-dependent transcripts such as *rpoB, rpoC, rpoC2* (encoding three subunits of PEP) were obviously up-regulated and the expression of the genes dependent on both PEP and NEP such as *16S rRNA* and *23S rRNA*, two components of the plastid translation machinery, declined sharply (Figure 7). In addition, the expression of *OsRpoTp* (encoding NEP core subunits, Hiratsuka et al. 1989) was increased, but the expression of *FtsZ* (encoding a component of the plastid division machinery, Takeuchi et al. 2007) was decreased significantly (Figure 7). Overall, the observations indicated that the *asl3* mutation affects the transcriptional expressions of genes associated with not only photosynthesis but also the early chloroplast development.

## Discussion

### *ASL3* encodes a chloroplast-targeted PPR protein which is necessary for the survival of rice

PPR genes constitute a large multigene family in higher plants. Recent studies have revealed that PPR proteins are essential for plant growth and development and most of them are involved in editing, splicing, and regulating the stability of various organellar transcripts (Schmitz-Linneweber and Small 2008). In contrast to *Arabidopsis* PPRs, very little is known about the functions of rice PPRs. Here, we present a molecular characterization of the PPR gene, rice *ASL3*, with 10 PPR motifs. The ASL3 protein was predicted to contain a chloroplast transit peptide (cTP) in its N-terminal region, suggesting that the protein is one of the PPRs targeted to chloroplast, and subcellular localization experiments confirmed this prediction. Similarly, few PPR genes that contain cTP were reported in rice such as *OsPPR1*, *YSA*, and *OsV4* (Gothandam et al. 2005; Su et al. 2012; Gong et al. 2014).

In this study, the lack of rice ASL3 leads to the albino seedling lethality and attributes to the hindrance of chloroplast development (Figure 2C, D) and Chl biosynthesis (Figure 1E). Furthermore, the *ASL3* RNAi transgenic lines were obtained with reduced expression of *ASL3* relative to WT plants and the albino phenotype was observed at early growth stages for *ASL3* RNAi lines (Figure 1C). These results show the importance of *ASL3* gene. In the previous studies, *osppr4* also showed an albino phenotype with early seedling lethality and the *OsPPR4* possesses 15 PPR motifs (Asano et al. 2013). The rice *osv4* mutant develops albino leaves initially at a restrictive low temperature (constant 20°C) but gradually turns green as the plants grow (Gong et al. 2014). Interestingly, the lack of homologs ASL3 in *Arabidopsis* leads to embryo lethality rather than albino seedling lethality (Cushing et al. 2005). This observation suggests that the functions of some PPR genes have changed during evolution in spite of the high conservative property. Taken together, these results suggest that the ASL3 is a chloroplast-targeted PPR protein which is essential for the survival of rice.

### *ASL3* may be involved in the regulation of early chloroplast development and plastid gene expression

The chloroplast is a semi-autonomous organelle, which contains about 100 genes, although more than 3,000 proteins function within it (Leister, 2003). Thus, nucleus-encoded factors play essential roles in the regulation of chloroplast development, which requires the coordinated expression of both nucleus-encoded and chloroplast-encoded genes. The processes accompanying chloroplast development can be divided into three steps in higher plants (Mullet 1993; Kusumi et al. 2010). The first step involves the proplastid growth and activation of plastid DNA synthesis. The second step is the chloroplast ‘build-up’ step, which is characterized by the establishment of transcription/translation apparatus. At this step, NEP preferentially transcribes plastid genes that encode elements of the transcription and translation apparatus (Hajdukiewicz et al. 1997) and the transcription and translation activity in the chloroplast is dramatically elevated. The final step is the high level expression of plastid and nuclear genes encoding photosynthetic apparatus. In particular, the plastid genes are exclusively transcribed by PEP (De Santis-MacIossek et al. 1999).

In *asl3* plants, the mutation disrupts the transcripts of plastid and nuclear genes associated with chloroplast development (Figure 7). The suppression on the *FtsZ* transcripts resulted in the less number of chloroplasts (Figure 2C), because it is essential in the first step of chloroplast development. Besides, the transcripts for NEP component (*OsRpoTp*) and PEP components (*rpoB*, *rpoC1* and *rpoC2*) accumulated to a high level, probably caused by feedback mechanism (Figure 7). However, transcript accumulation of both PEP- and NEP- dependent genes(*16S rRNA*, *23S rRNA*) and PEP-transcribed plastid genes (*psbA*, *rbcL*) were severely suppressed (Figure 7), suggesting that, in *asl3* mutant, accumulation of transcripts for PEP components did not result in the formation of functional PEP due to the disruption of transcription/translation apparatus. Similar conclusions were also obtained in maize *ppr2* mutant considering that *PPR2* functions in the synthesis or assembly of one or more component of the plastid translation machinery (Williams and Barkan 2003). In addition, the plastid-to-nucleus signaling pathways in *asl3* mutant probably were changed and finally affected the expressions of nuclear-encoded genes required for photosynthesis (*Cab1R* and *RbcS*).This result was in accordance with the previous results from *v2* mutant (Sugimoto et al. 2004) and another rice albino mutant, *asl1* (Gong et al. 2013).

Most PPR proteins are involved in editing, splicing, and regulating the stability of various organellar transcripts (Schmitz-Linneweber and Small 2008). However, those evidences are mainly obtained in *Arabidopsis* research but rarely obtained in rice. Asano et al. (2013) reported that OsPPR4 is required for splicing of chloroplast transcripts and RNA editing of *ndhA*. Disruption of *OsPPR4* expression led to a strong defect in the splicing of *atpF*, *ndhA*, *rpl2*, and *rps12-2* introns and influences the splicing of *petB* and *rps16* introns. The rice DYW-class PPR protein, OGR1, is essential for RNA editing in rice mitochondria and is required for normal growth and development (Kim et al. 2009). In this study, although specific target RNA has not been found yet, our results still reveal some useful information. For example, transcript levels of some ribosomal components and PEP-dependent genes are dramatically reduced in the albino mutants. Furthermore, our study with the antisense plant demonstrated that the *ASL3* gene plays an important role in the early chloroplast development of rice. Probably, the *ASL3* gene is involved in the processing of plastid RNA required for the early event of chloroplast biogenesis. Further genetic and biochemical studies of *ASL3* will be required to gain insight into its detailed function.

## Conclusion

The *ASL3* gene encoded a novel chloroplast-targeted PPR protein with 10 tandem PPR motifs in rice. Disruption of the ASL3 would lead to a defective chloroplast and seedling lethality, and affected expression levels of genes associated with chloroplast development and photosynthesis at early leaf stage of rice.

## Methods

### Plant materials and growth conditions

The rice albino mutant *asl3* used in this study was isolated from a ^60^Co gamma rays irradiated mutant pool of *Oryza sativa* cultivar Jiahua1 (WT, *japonica* rice variety). To generate a large F_2_ populations for genetic studies, crosses were conducted between heterozygous plants (*ASL3*/*asl3*) and an indica cultivar Pei’ai64S. For phenotypic characterization, pigment content measurement and RNA extraction, seeds of the WT and *asl3* plants were grown in growth chambers under controlled 12 h of light and 12 h of dark at a constant temperature of 32°C and humidity of approximately 70%. The *asl3* mutants can be distinguished from the normal segregants by albino phenotype.

### Cloning of *ASL3*

To map the *ASL3* gene, 22 individuals with typical albino phenotype were screened out from an F_2_ populations derived from a cross between the heterozygous plants (*ASL3*/ *asl3)* and Pei’ai64S for linkage analysis. Then a total of 4213 F_2_ mutant individuals were selected for fine-mapping. Genomic DNA was extracted from young leaves by the CTAB method and analyzed for cosegregation using available simple sequence repeat markers (McCouch et al. 2002). New insertion-deletion (InDel) markers were developed based on the entire genomic sequences of Nipponbare variety (Goff et al. 2002) and *indica* variety 93–11 (Yu et al. 2002). The sequences of the markers were designed using the PREMIRE5.0 software. The markers are listed in Additional file 1: Table S1. Gene prediction was performed using the Rice Genome Annotation Project (http://rice.plantbiology.msu.edu/cgi-bin/gbrowse/rice/). The genomic DNA fragments of candidate genes from the mutant and WT plants were amplified and sequenced.

### RNAi suppression of *ASL3*

To confirm that *ASL3* was the gene associated with the phenotype observed, RNA interference (RNAi) analysis was preformed. The construct vector pTCK303 with a maize ubiquitin promoter and a rice intron was used as an RNAi vector (Wang et al. 2004). Both anti-sense and sense versions of a specific 414-bp fragment from the coding region of the *ASL3* were amplified, and successively inserted into pTCK303, to form the RNAi construct vector pTCK303-dsRNAiASL3. The primer pairs are 5’ CGAGCTCGGTTGGAACCAGACCTCATTGTG 3’, 5’ GACTAGTCACCTTGCAAATCTTCTCGACCT 3’ and 5’ CGGGATCCCGGTTGGAACCAGACCTCATTGTG 3’, 5’ GGGGTACCCCACCTTGCAAATCTTCTCGACCT 3’. Then, the resultant plasmid and the empty vector were introduced into *Agrobacterium* tumefaciens EHA105 and then used to infect calli of WT plants according to a published method (Hiei et al. 1994).

### Chlorophyll and carotenoid content measurement

Both chlorophyll (Chl) and carotenoid (Car) contents of the 3-leaf-stage leaves were measured following the method of Arnon (1949). Briefly, leaves (approximately 0.02 g fresh weight) were cut and marinated in 5 ml of 5:4:1 acetone: ethanol: H_2_O for 18 h under dark conditions. Residual plant debris was removed by centrifugation. The supernatants were analyzed with a DU 800 UV/Vis Spectrophotometer (Beckman Coulter) at 665, 649 and 470 nm, respectively.

### Transmission electron microscopy (TEM) analysis

For TEM analysis, the transverse sections of top leaves sampled from the 3-leaf-stage WT and *asl3* seedlings grown in a growth chamber at 32°C were fixed in a solution of 2.5% glutaraldehyde and then fixed in 1% OsO4. After staining with uranyl acetate, tissues were further dehydrated in an ethanol series and finally embedded in Spurr’s medium prior to ultrathin sectioning. Samples were stained again and examined with a Hitachi-7650 transmission electron microscope.

### Phylogenetic analysis

Homologous sequences of ASL3 were identified using the Blast search program of the National Center for Biotechnology Information (NCBI, http://www.ncbi.nlm.nih.gov/). The functional domain analysis was performed TPRpred (Karpenahalli et al. 2007). The sequences of PPR domains were aligned using BioXM version 2.6 software and the neighbor-joining tree was generated with the Poisson correction method with MEGA version 5.1 software. Bootstrap replication (1000 replications) was used for a statistical support for the nodes in phylogenetic tree.

### Subcellular localization

To investigate the subcellular localization of ASL3, the cDNA fragment encoding the N-terminal region (amino acids 1–249) of the ASL3 was amplified from WT plants using primer pair 5’GGAAGATCTTGTGTGTGTGTGTGTGATG3’, 5’CGGGGTACCAAATGAGCAACCATACTACC3’ and introduced into vector pMON530-GFP at the *Kpn*I and *Bgl*II sites. Transformation was performed according to the method of Yin et al. (2012). The GFP fluorescences of the transgenic tobacco (*Nicotiana tabacum*) cells were observed under Confocal Laser Scanning Microscopy (LSM 5 PASCAL; ZEISS, http://www.zeiss.com). The GFP fluorescence images were obtained using an argon ion laser with excitation at 488 nm and a 505–530 nm band-pass filter. Chlorophyll autofluorescence was detected with a 570-nm filter.

### RT-PCR and quantitative real-time PCR (qRT-PCR) analysis

Total RNA was extracted from seedling roots, young stems, young leaves, flag leaves and young panicles using an RNA Prep Pure Plant kit (Tiangen Co., Beijing, China). For RT-PCR, first-strand cDNA was reverse transcribed from total RNA with RT primer mix (oligo dT and random 6 mers). Real-time PCR was performed using a SYBR_ Premix Ex TaqTM kit (TaKaRa) on an ABI prism 7900 Real-Time PCR System. The 2^-ΔΔC^T method was used to analyze the relative changes in gene expression (Livak and Schmittgen 2001). The primers for photosynthesis and chloroplast development associated genes (*FtsZ*, *OsRpoTp*, *rpoB*, *rpoC1*, *rpoC2*, *Cab1R*, *rbcS*, *RbcL*, *psbA*, *16S rRNA*, *23S rRNA*) were listed in Additional file 2: Table S2. The rice *Actin* gene was used as a reference gene in this study.

## Additional files

Additional file 1: Table S1. PCR-based molecular markers designed for fine mapping. Additional file 2: Table S2. Markers designed for Real-time PCR.
