# Supplementary material for: The rice ALS3 encoding a novel pentatricopeptide repeat protein is required for chloroplast development and seedling growth
Source: Rice (N Y). 2015 Apr 9;8:17. doi: 10.1186/s12284-015-0050-9 (PMC4390607; doi:10.1186/s12284-015-0050-9)
Supplement: Additional file 1: Table S1. — PCR-based molecular markers designed for fine mapping. [file 12284_2015_50_MOESM1_ESM.ppt]

## Slide 1
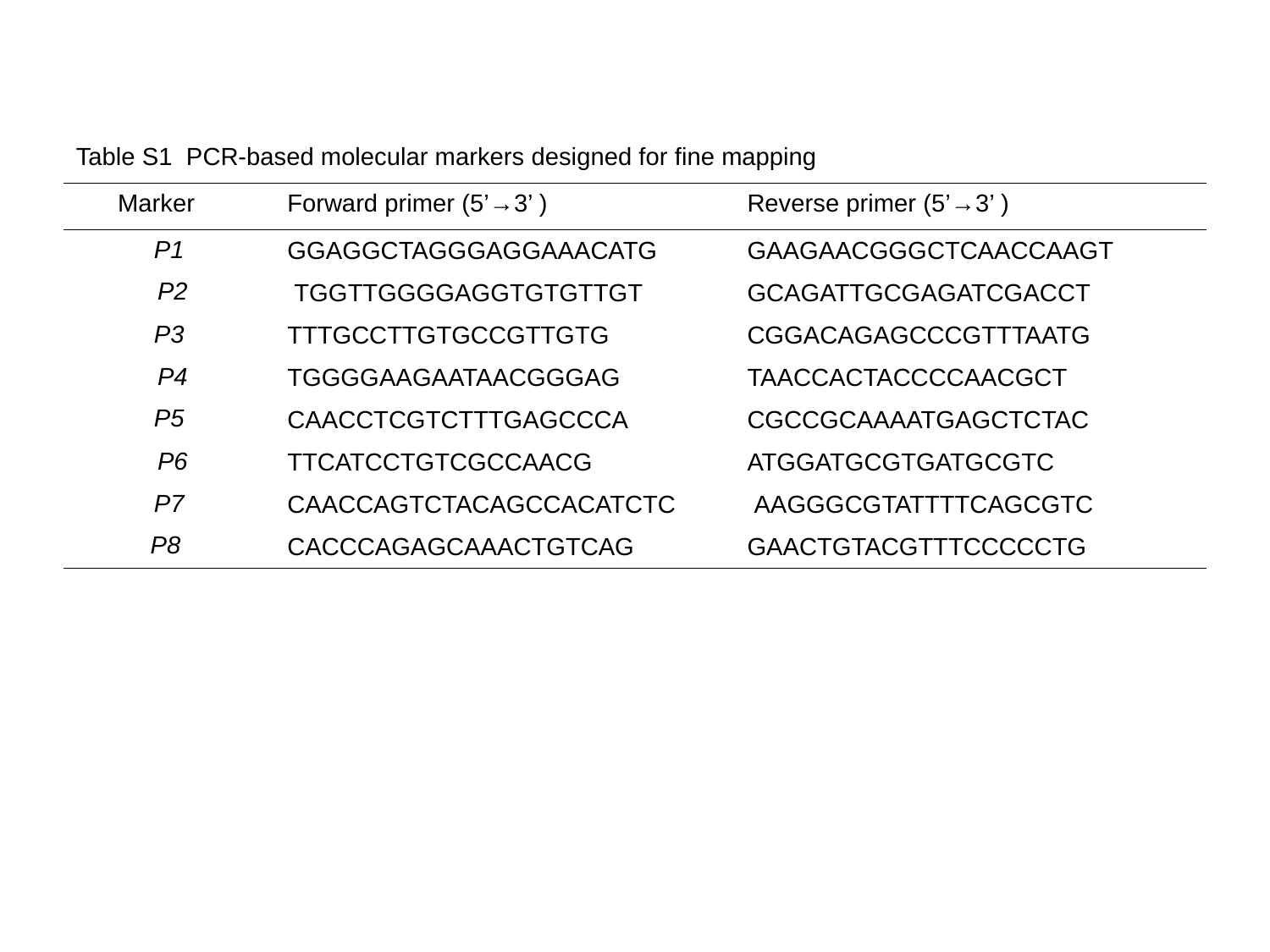

| Table S1 PCR-based molecular markers designed for fine mapping | | |
| --- | --- | --- |
| Marker | Forward primer (5’→3’ ) | Reverse primer (5’→3’ ) |
| P1 | GGAGGCTAGGGAGGAAACATG | GAAGAACGGGCTCAACCAAGT |
| P2 | TGGTTGGGGAGGTGTGTTGT | GCAGATTGCGAGATCGACCT |
| P3 | TTTGCCTTGTGCCGTTGTG | CGGACAGAGCCCGTTTAATG |
| P4 | TGGGGAAGAATAACGGGAG | TAACCACTACCCCAACGCT |
| P5 | CAACCTCGTCTTTGAGCCCA | CGCCGCAAAATGAGCTCTAC |
| P6 | TTCATCCTGTCGCCAACG | ATGGATGCGTGATGCGTC |
| P7 | CAACCAGTCTACAGCCACATCTC | AAGGGCGTATTTTCAGCGTC |
| P8 | CACCCAGAGCAAACTGTCAG | GAACTGTACGTTTCCCCCTG |
