# Supplementary material for: The rice ALS3 encoding a novel pentatricopeptide repeat protein is required for chloroplast development and seedling growth
Source: Rice (N Y). 2015 Apr 9;8:17. doi: 10.1186/s12284-015-0050-9 (PMC4390607; doi:10.1186/s12284-015-0050-9)
Supplement: Additional file 2: Table S2. — Markers designed for Real-time PCR. [file 12284_2015_50_MOESM2_ESM.ppt]

## Slide 1
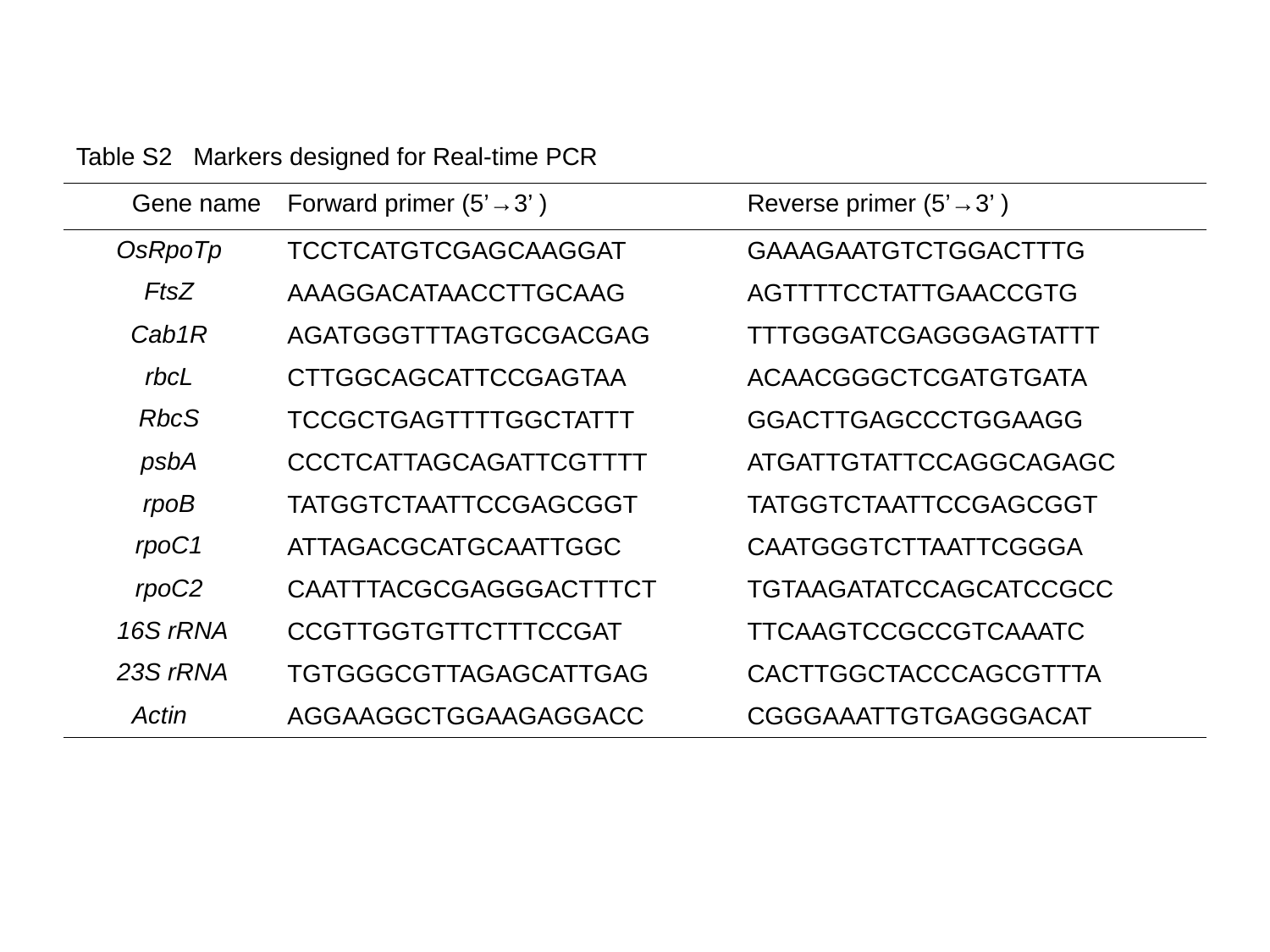

| Table S2 Markers designed for Real-time PCR | | |
| --- | --- | --- |
| Gene name | Forward primer (5’→3’ ) | Reverse primer (5’→3’ ) |
| OsRpoTp | TCCTCATGTCGAGCAAGGAT | GAAAGAATGTCTGGACTTTG |
| FtsZ | AAAGGACATAACCTTGCAAG | AGTTTTCCTATTGAACCGTG |
| Cab1R | AGATGGGTTTAGTGCGACGAG | TTTGGGATCGAGGGAGTATTT |
| rbcL | CTTGGCAGCATTCCGAGTAA | ACAACGGGCTCGATGTGATA |
| RbcS | TCCGCTGAGTTTTGGCTATTT | GGACTTGAGCCCTGGAAGG |
| psbA | CCCTCATTAGCAGATTCGTTTT | ATGATTGTATTCCAGGCAGAGC |
| rpoB | TATGGTCTAATTCCGAGCGGT | TATGGTCTAATTCCGAGCGGT |
| rpoC1 | ATTAGACGCATGCAATTGGC | CAATGGGTCTTAATTCGGGA |
| rpoC2 | CAATTTACGCGAGGGACTTTCT | TGTAAGATATCCAGCATCCGCC |
| 16S rRNA | CCGTTGGTGTTCTTTCCGAT | TTCAAGTCCGCCGTCAAATC |
| 23S rRNA | TGTGGGCGTTAGAGCATTGAG | CACTTGGCTACCCAGCGTTTA |
| Actin | AGGAAGGCTGGAAGAGGACC | CGGGAAATTGTGAGGGACAT |
